# A homozygous MED11 C-terminal variant causes a lethal neurodegenerative disease

**DOI:** 10.1016/j.gim.2022.07.013

**Published:** 2022-10

**Authors:** Elisa Calì, Sheng-Jia Lin, Clarissa Rocca, Yavuz Sahin, Aisha Al Shamsi, Salima El Chehadeh, Myriam Chaabouni, Kshitij Mankad, Evangelia Galanaki, Stephanie Efthymiou, Sniya Sudhakar, Alkyoni Athanasiou-Fragkouli, Tamer Çelik, Nejat Narlı, Sebastiano Bianca, David Murphy, Francisco Martins De Carvalho Moreira, SYNaPS Study Group, Michael G. Hannah, Enrico Bugiardini, Yamna Kriouile, Mohamed El Khorassani, Mhammed Aguennouz, Stanislav Groppa, Blagovesta Marinova Karashova, Gabriella Di Rosa, Jatinder S. Goraya, Tipu Sultan, Daniela Avdjieva, Hadil Kathom, Radka Tincheva, Selina Banu, Pierangelo Veggiotti, Alberto Verrotti, Salvatore Savasta, Alfons Macaya Ruiz, Barbara Garavaglia, Eugenia Borgione, Savvas Papacostas, Chiara Compagnoni, Alessandra Piccirilli, Michail Vikelis, Viorica Chelban, Rauan Kaiyrzhanov, Andrea Cortese, Roisin Sullivan, Eleni Zamba Papanicolaou, Efthymios Dardiotis, Shazia Maqbool, Shahnaz Ibrahim, Salman Kirmani, Nuzhat Noureen Rana, Osama Atawneh, Shen-Yang Lim, Farooq Shaikh, Annarita Scardamaglia, George Koutsis, Salvatore Mangano, Carmela Scuderi, Eugenia Borgione, Giovanna Morello, Massimo Zollo, Gali Heimer, Pasquale Striano, Issam Al-Khawaja, Fuad Al-Mutairi, Fowzan S. Alkuraya, Mie Rizig, Chingiz Shashkin, Nazira Zharkynbekova, Kairgali Koneyev, Ganieva Manizha, Maksud Isrofilov, Ulviyya Guliyeva, Kamran Salayev, Samson Khachatryan, Georgia Xiromerisiou, Cleanthe Spanaki, Arianna Tucci, Chiara Fiorillo, Federico Rissotto, Francina Munell, Antonella Gagliano, Farida Jan, Roberto Chimenz, Eloisa Gitto, Caterina Cuppari, Carmelo Romeo, Francesca Magrinelli, Neerja Gupta, Madhulika Kabra, Hanene Benrhouma, Meriem Tazir, Luca Zagaroli, Claudia Caloisi, Cecilia Fabiano, Gabriella Bottone, Giovanni Farello, Sandra Di Fabio, Makram Obeid, Sophia Bakhtadze, Nebal W. Saadi, Maha S. Zaki, Chahnez C. Triki, Majdi Kara, Vincenzo Belcastro, Nicola Specchio, Ehsan G. Karimiani, Ahmed M. Salih, Luca A. Ramenghi, Emanuele David, Riccardo Curró, Maria Laura Iezzi, Giulia Iapadre, Giuliana Nanni, Giovanna Scorrano, Maria F. Fiorile, Francesco Brancati, Giovanna Di Falco, Luana Mandarà, Giuseppe Barrano, Maurizio Elia, Gaetano Terrone, Francesca F. Operto, Mariella Valenzise, Ylenia Della Rocca, Francesca Zazzeroni, Edoardo Alesse, Filippo Manti, Serena Galosi, Francesca Nardecchia, Vincenzo Leuzzi, Erica Pironti, Greta Amore, Giorgia Ceravolo, Faisal Zafar, Ehsan Ullah, Erum Afzal, Iram Javed, Fatima Rahman, Muhammad Mehboob Ahmed, Pasquale Parisi, Paola Borgia, Giuseppe D. Mangano, Francesco Chiarelli, Queen Square Genomics, Andrea Accogli, Cassidy Petree, Kevin Huang, Kamel Monastiri, Masoud Edizadeh, Rosaria Nardello, Marzia Ognibene, Patrizia De Marco, Martino Ruggieri, Federico Zara, Pasquale Striano, Yavuz Şahin, Lihadh Al-Gazali, Marie Therese Abi Warde, Benedicte Gerard, Giovanni Zifarelli, Christian Beetz, Sara Fortuna, Miguel Soler, Enza Maria Valente, Gaurav Varshney, Reza Maroofian, Vincenzo Salpietro, Henry Houlden

**Affiliations:** 1 Department of Neuromuscular Diseases, UCL Queen Square Institute of Neurology, London, United Kingdom; 2 Genes & Human Disease Research Program, Oklahoma Medical Research Foundation, Oklahoma City, OK; 3 Department of Medical Genetics, Genoks Genetic Laboratory, Ankara, Turkey; 4 Tawam Hospital, Al Ain, United Arab Emirates; 5 Service de Génétique Médicale, Institut de Génétique Médicale d’Alsace (IGMA), Centre de Référence des Déficiences Intellectuelles de Causes Rares, Centre de Recherche en Biomédecine de Strasbourg (CRBS), Hôpitaux Universitaires de Strasbourg, Strasbourg, France; 6 LGC genetics, Centre Medical les Jasmins, Tunis, Tunisia; 7 Department of Radiology, Great Ormond Street Hospital for Children, London, United Kingdom; 8 Department of Pediatric Neurology, Private Practice, Adana, Turkey; 9 Division of Neonatology, Department of Pediatrics, Medical School, Cukurova University, Adana, Turkey; 10 Medical Genetics, Referral Centre for Rare Genetic Diseases, ARNAS Garibaldi, Catania, Italy; 11 Department of Health Promotion, Mother and Child Care, Internal Medicine and Medical Specialities “G. D'Alessandro,” University of Palermo, Palermo, Italy; 12 Department of Clinical and Experimental Epilepsy, UCL Institute of Neurology, Queen Square, London, United Kingdom; 13 Division of Medical Genetics, Department of Specialized Medicine, Montreal Children's Hospital, McGill University Health Centre (MUHC), Montreal, Quebec, Canada; 14 Department of Human Genetics, McGill University, Montreal, Quebec, Canada; 15 Department of Neonatology, Fattouma Bourguiba Hospital, Monastir, Tunisia; 16 Unit of Medical Genetics, IRCCS Istituto Giannina Gaslini, Genoa, Italy; 17 Unit of Rare Diseases of the Nervous System in Childhood, Department of Clinical and Experimental Medicine, Section of Pediatrics and Child Neuropsychiatry, AOU “Policlinico,” University of Catania, Catania, Italy; 18 Department of Neurosciences, Rehabilitation, Ophthalmology, Genetics, Maternal and Child Health, University of Genoa, Genoa, Italy; 19 Pediatric Neurology and Muscular Diseases Unit, IRCCS “Istituto Giannina Gaslini,” Genova, Italy; 20 Department of Medical Genetics, Biruni University, İstanbul, Turkey; 21 Department of Pediatrics, College of Medicine and Health Sciences, United Arab Emirates University, Al Ain, United Arab Emirates; 22 Pediatric Neurology Department, CHU Strasbourg, Strasbourg, France; 23 Laboratoires de Diagnostic Génétique, Unité de Génétique Moléculaire, Nouvel Hôpital Civil, Strasbourg Cedex, France; 24 CENTOGENE AG, Rostock, Germany; 25 Department of Chemical and Pharmaceutical Sciences, University of Trieste, Trieste, Italy; 26 CONCEPT Lab, Italian Institute of Technology (IIT), Genova, Italy; 27 Department of Molecular Medicine, University of Pavia, Pavia, Italy; 28 Neurogenetics Research Center, IRCCS Mondino Foundation, Pavia, Italy; 29 Department of Pediatrics, University of L’Aquila, L’Aquila, Italy

**Keywords:** Human mediator complex, *MED11*, MEDopathies

## Abstract

**Purpose:**

The mediator (MED) multisubunit-complex modulates the activity of the transcriptional machinery, and genetic defects in different MED subunits (17, 20, 27) have been implicated in neurologic diseases. In this study, we identified a recurrent homozygous variant in *MED11* (c.325C>T; p.Arg109Ter) in 7 affected individuals from 5 unrelated families.

**Methods:**

To investigate the genetic cause of the disease, exome or genome sequencing were performed in 5 unrelated families identified via different research networks and Matchmaker Exchange. Deep clinical and brain imaging evaluations were performed by clinical pediatric neurologists and neuroradiologists. The functional effect of the candidate variant on both MED11 RNA and protein was assessed using reverse transcriptase polymerase chain reaction and western blotting using fibroblast cell lines derived from 1 affected individual and controls and through computational approaches. Knockouts in zebrafish were generated using clustered regularly interspaced short palindromic repeats/Cas9.

**Results:**

The disease was characterized by microcephaly, profound neurodevelopmental impairment, exaggerated startle response, myoclonic seizures, progressive widespread neurodegeneration, and premature death. Functional studies on patient-derived fibroblasts did not show a loss of protein function but rather disruption of the C-terminal of MED11, likely impairing binding to other MED subunits. A zebrafish knockout model recapitulates key clinical phenotypes.

**Conclusion:**

Loss of the C-terminal of MED subunit 11 may affect its binding efficiency to other MED subunits, thus implicating the MED-complex stability in brain development and neurodegeneration.

## Introduction

Mediator (MED) is an evolutionarily conserved multisubunit protein complex composed of 25 to 30 distinct proteins grouped in 4 modules: head, middle, tail (core complex), and a separable regulatory Cdk8 kinase module. It plays an essential role in several transcription processes, acting as a functional bridge between transcription factors and the basal transcriptional machinery.^1^^,^^2^ Although presumed that MED complex acts as a single unit, genetic defects of different subunits of the complex result in distinct disorders with overlapping clinical features, implying that either parts of the complex have separate functions or MED proteins have functions (outside of the complex) that are specific to each subunit.^2^, ^3^, ^4^, ^5^, ^6^, ^7^ Particularly, pathogenic (biallelic) variants in subunits part of the MED head module (eg, *MED17*, OMIM 613668; *MED20*, OMIM 612915; *MED27*, OMIM 619286) have been implicated in rare neurologic disorders characterized by severe neurodevelopmental impairment, congenital and/or postnatal microcephaly, and variable degrees of progressive central nervous system (CNS) degeneration (Supplementary Figure 1).^8^, ^9^, ^10^ We hereby delineate a new autosomal recessive neurodegenerative disorder caused by a recurrent homozygous truncating variant in *MED11*, encoding a subunit portion of the head module involved in the MED-complex stability.^1^^,^^2^^,^^11^ All the affected individuals exhibit congenital microcephaly, profound global developmental delay, frequent refractory myoclonic seizures, movement disorder with exaggerated startle responses, and prenatal-onset neurodegeneration with severely progressive CNS atrophy.

## Materials and Methods

The affected individuals were identified by screening genomic data sets from several diagnostic and research genetic laboratories internationally, as well as using GeneMatcher.^12^ After obtaining signed informed consent forms, clinical data and DNA samples were collected from participating families and used under research project approved by the Review Boards and Bioethics Committees at University College London Hospital (project 06/N076) and the other participating institutions. Either exome sequencing or genome sequencing was performed at different diagnostic or research laboratories as described elsewhere.^13^^,^^14^ The candidate variants were confirmed after filtering and interpretation according to the American College of Medical Genetics and Genomics/Association for Molecular Pathology guidelines,^15^ and segregation analyses were carried out using Sanger sequencing. Semiquantitative reverse transcriptase polymerase chain reaction (RT-PCR) and western blotting were performed with extracted protein and RNA from fibroblasts derived from affected individual A.II.1 and his unaffected mother. Homozygosity mapping and haplotype analysis were performed on the genetic data from 3 affected individuals (A.II.1, C-II.1, and D.II.1), 2 parents, and 5 unrelated healthy controls age- and ethnically matched.^16^ The human MED structure resolved by cryogenic electron microscopy was retrieved from the article by Rengachari et al^17^ and analyzed to assess the computational effect of the variant on the MED-complex head module stability and dynamics. The whole-mount RNA in situ hybridization was performed to assess mRNA expression, and knockouts in zebrafish were generated using clustered regularly interspaced short palindromic repeats/Cas9 using 2 single-guide RNAs targeting the exon 3 of *med11* according to methods described earlier.^18^ Phenotype analysis was performed on 5 days post fertilization (dpf) animals. Animals were placed on an agarose cavity and images were taken using a stereomicroscope. The head and eye sizes were measured from scale-calibrated images using ImageJ software (National Institutes of Health). Visual startle response and auditory evoked behavior response was performed using DanioVision (Noldus) and zebrabox (ViewPoint) monitoring systems as described previously.^18^

## Results

**Figure 1:**
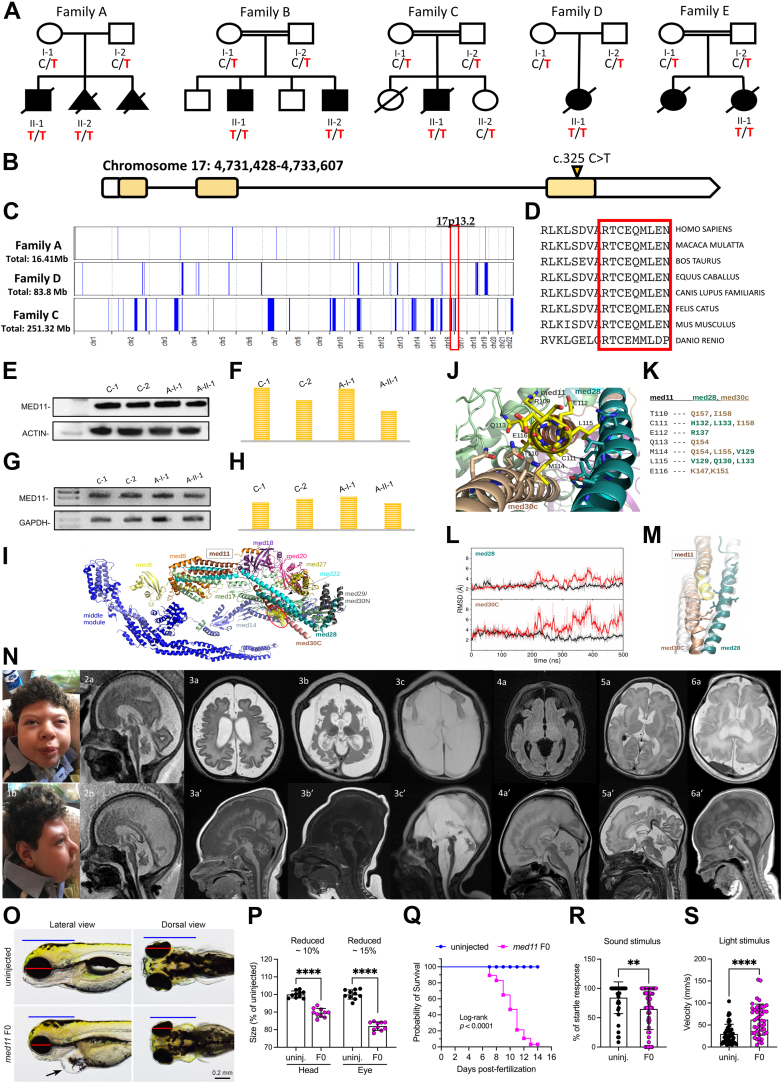
**Clinical, molecular and neuroradiological features of the affected individuals.** A. Pedigrees of affected families. Solid black indicates affected. Genotype, where indicated, represent results of evaluation for the MED11 c.325C>T variant using Sanger sequencing. B. Structure of the MED11 gene with the variant. C. Overview of the whole regions of homozygosity (ROH) in the exome of each case from 3 families. The region of homozygosity surrounding the MED11 variant is indicated in red bracket (the homozygous variant in family A is not within an ROH). D. Conservation of the C-terminal residue of MED11 protein through different species. E. Western blotting from protein extracted from fibroblast cell lines of 1 proband, the heterozygous parent, and 2 wild-type age-matched controls. F. Analysis of the western blot using the densitometry software ImageJ after normalization relative to a housekeeping protein (actin) and calculation using a relative relationship method. G. Reverse transcription polymerase chain reaction (PCR) amplified mutant complementary DNA from messenger RNA extracted from fibroblast cell lines of 1 proband, the heterozygous parent, and 2 wild-type age-matched controls. H. Analysis of the semiquantitative PCR using the densitometry software ImageJ after normalization relative to a housekeeping gene (GAPDH) and calculation using a relative relationship method. I. Structure of the human mediator. The med11 C-terminal region affected by the nonsense variant at R109 is highlighted by a yellow surface. J. Detailed representation of the med11 C-terminal region within the med11-med28-med30C bundle structure. K. List of interactions between med11 C-terminal region and med28, med30C residues. L. Root mean square deviation values of med28 and med30C residues that interact with med11 C-terminal were calculated for the wild type (black) and the mutant (red) after med11 alignment. M. Comparison between the most representative structure of mutant med11, med28, and med30C, obtained as a medoid of the biggest cluster of the trajectory, and the crystal structure (in transparent white). N. Facial appearance of individual A-II-1 (1a and b) and representative brain abnormalities on magnetic resonance image. Individual A-II-1: progressive global neurodegeneration and atrophy involving the cerebral and cerebellar hemispheres shown at age 1 month (3a: axial T2, 3a′: sagittal T1), at 4 months (3b: axial T2, 3b′: sagittal T1), and 2 years (3c: axial T2, 3c′: sagittal T2). Note was also made of cerebral dysgyria secondary to immature cortical folding. Bilateral subdural effusions were noted secondary to the severe atrophy. Diffuse white-matter immaturity was also noted—delayed-/hypo-myelination therefore could not be excluded. Another feature noted was severe degeneration of the basal ganglia structures. Fetal scans (of the aborted fetus) at 31 weeks (2a: sagittal T2) and at 34 weeks of gestation (2b: sagittal T2) again show progressive atrophy of intracranial structures with increasing extra-axial spaces, cerebral dysgyria, and marked cerebellar atrophy. Individual B-II-1 (4a: axial FLAIR, 4a′: sagittal T2) and individual B-II-2, (5a: axial T2, 5a′: sagittal T2) showed similar features of global brain underdevelopment with cerebral dysgyria and particular cerebellar atrophy; 6a: axial T2 and 6a′: sagittal T1 images in the same affected individual showing similar features of underopercularization with cortical dysgyria, immaturity and underdevelopment of the white matter, and cerebellar atrophy. O. Morphologic phenotyping of *med11* knockout animals. Control animals are shown in top panel, *med11* knockout animals show small brain and small eyes; red line represents the eye diameter and blue line shows brain size. Black arrow shows heart edema. P. Quantification of head and eye size. Q. Kaplan-Meier survival curves, time shown in days, the log rank test was used for statistical analysis. R. Auditory evoked behavior response analysis of knockout animals showed reduced startle response. S. Visual startle response analysis showed increased movement after the light stimulus.

A total of 7 affected individuals were identified from 5 unrelated families. The clinical features of each individual are summarized in Table 1 and shown in Figure 1. Consanguinity was reported in 3 families. All children presented at birth with severe respiratory distress requiring intubation. Four individuals died because of cardiorespiratory insufficiency in their early infancy, and 2 required prolonged assisted ventilation through their childhood. Distinctive craniofacial features, including trigonocephaly and frontal bossing, were noticed in all the affected individuals (Figure 1E). Congenital microcephaly was recorded in 4 affected individuals. All subjects presented global developmental delay, ranging from severe to profound, with lack of achievement of any developmental milestones. Seizures or electroencephalogram (EEG) abnormalities were observed in all children. In individual B-II-2, EEG documented an encephalopathic pattern with low amplitude attenuated background of mixed delta and theta waves. Individual D-II-1 did not present with seizures, but EEG showed burst suppression pattern and discontinuous tracing, consistent with a diagnosis of developmental encephalopathy. In the remaining affected children (A-II-1, B-II-1, C-II-1, E-II-1), onset of epilepsy was within the first month of life, with myoclonic seizures refractory to treatment in all individuals. Hyperkinetic (hyperekplexia-like) movement disorders characterized by exaggerated startle responses after tactile stimulations were also exhibited by most cases; additional movement abnormalities included tremor and trismus. Neurologic examination was remarkable for axial hypotonia and upper and/or lower limb hypertonia in all affected individuals. Other findings included impaired hearing with congenital bilateral hearing loss and vision abnormalities with nystagmus and/or strabismus and bilateral optic atrophy. Brain magnetic resonance imaging showed CNS atrophy with increasing extra-axial spaces and immature cortical folding with cortical dysgyria (individual A-II-2) since prenatal life, and postnatal magnetic resonance imaging (individuals A-II-1, B-II-1, B-II-2) in different families documented severe progression of the global atrophy involving the cerebral and, particularly, the cerebellar hemispheres as well as diffuse white-matter immaturity with evidences of hypomyelination; degeneration of the basal ganglia structures was also noticed at follow-up imaging in individual A-II-1. Individual A-II-2 was terminated in utero at 34th gestational week owing to the identification of CNS anomalies and DNA extracted from the aborted fetus was conserved for genetic testing. All affected individuals recruited in this study underwent extensive genetic testing, including single-gene and microcephaly and/or epilepsy panels screening, before undergoing exome or genome sequencing. Interestingly, all 7 individuals were found to have the same ultrarare homozygous variant in *MED11* (Chr17-4636453-C-T; NM_001001683.4: c.325C>T) segregating with the disease across the 5 unrelated families. No other pathogenic, likely pathogenic, or variant of significance in genes associated with neurodevelopmental disorders were found upon exome or genome sequencing in the affected individuals. After screening different international genomic data sets (including in-house database, Genome Aggregation Database [https://gnomad.broadinstitute.org] data set, UK Biobank and CENTOGENE database), we observed the *MED11* variant in heterozygous state in only 14 individuals out of >500,000 exomes/genomes. Detailed information on the variant is reported in Supplemental Table 1. The variant was residing within a sizable region of homozygosity in family C and D, whereas it was not within any region of homozygosity in family A (Figure 1C). Haplotype analysis showed distinct haplotypes on the basis of single-nucleotide variations (formerly single-nucleotide polymorphism [SNP]) retrieved from exome sequencing data. The homozygous MED11 p.Arg109Ter variant leads to a premature truncation of the protein at amino acid position 109 and the loss of the last 9 conserved residues. As expected, western blot data and semiquantitative RT-PCR indicated that the truncated MED11 escaped nonsense-mediated decay, because MED11 protein and transcript expression levels in individual A.II.1 were similar to those of healthy heterozygotes and healthy controls (Figure 1E-H). Computational studies and simulations indicated that the recurrent truncating variant affects the interactions among the 3 helices of the subunits MED11, MED28, and MED30C. The MED11 C-terminal region harboring the p.Arg109Ter variant (Figure 1I) interacts mainly with the residues of MED28 and MED30C alpha-helical structures by forming a bundle-type hydrophobic structure together with MED22 (Figure 1J and K). The same residues observed to form a hydrophobic patch with MED11 C-terminal in the wild-type system significantly modify the conformation of the helix-bundle domain in the proximal tail in R109 (Figures 1L and M), suggesting that the 4-helix bundle formed by MED11, MED22, MED28, and MED30C has a role in the correct functionality and stability of the MED complex, consistently with previous studies.^19^^,^^20^

**Table 1:** Clinical features of patients with homozygous p. Arg109Ter variant

Family	Family A		Family B		Family C	Family D	Family E
	A-II-1	A-II-2	B-II-1	B-II-2	C-II-1	D-II-1	E-II-1
Ethnicity	South Italian	South Italian	Saudis/Yemenis	Saudis/Yemenis	Turkish	Tunisian	Tunisian
Sex	M	F	M	M	M	F	F
Age at last follow up	Died at 6 y	34th wk of gestation	6 y	2 mo	8 mo	Died at 10 d	Died 24 h after birth
IUGR	+	+	–	–	–	+	–
Gestational age at birth, wk	41	n/a	34	34	38	38	40
Head circumference, (at birth and follow up)	32 cm, 38 cm at 3y	n/a	29 cm, 34 cm at 2y	30.5 cm, 31.5 at 2 mo	n/a	n/a	n/a
Respiratory failure at birth	+	n/a	+	+	+	+	+
Tone	Axial hypotonia, appendicular hypertonia	n/a	Hypotonia	Hypotonia	Axial hypotonia, lower limb hypertonia	Axial hypotonia, lower limb hypertonia	Appendicular hypertonia
Global developmental delay	+	n/a	+	n/a	+	n/a	n/a
Seizures, type, onset	Yes, myoclonic, neonatal onset	n/a	Yes, myoclonic, neonatal onset	n/a	Yes, myoclonic, neonatal onset	n/a	Yes, myoclonic, neonatal onset
EEG (initial and follow up)	Slow cortical activity	n/a	Low amplitude, attenuated background of mixed delta theta	Low amplitude, attenuated background of mixed delta theta	Slow activity, diffuse arrhythmia, multifocal epileptic disorders	Areactive, discontinuous tracing, burst suppression pattern	n/a
Movement disorder	+	n/a	–	+	+	+	Trismus
	Exaggerated startle responses triggered by tactile stimulations, chewing movements			Exaggerated startle responses triggered by tactile stimulations	Exaggerated startle responses triggered by tactile stimulations	Fasciculation and tremors	
Brain MRI abnormalities	Progressive global atrophy involving the cerebral and cerebellar hemispheres, cerebral dysgyria, immature white matter, conspicuous basal ganglia degeneration	Progressive atrophy with particular cerebellar atrophy and dysgyria due to immature cortical folding	Global underdevelopment of the brain with cerebral dysgyria, particular cerebellar hypoplasia and atrophy	Cortical dysgyria, cerebellar atrophy, inferior cerebellar vermis hypoplasia, global cerebral underdevelopment with white-matter immaturity	Parenchymal atrophy with immature white matter	Supratentorial atrophy, cortical dysgyria, diffuse white matter immaturity	Cortical atrophy
Ophthalmological findings	Bilateral congenital cataract	n/a	Nystagmus	–	Nystagmus, strabismus	n/a	n/a
Bilateral hearing loss	+	n/a	+	+	n/a	n/a	n/a
Dysmorphic features	Triangular face, frontal bossing, occipital flattening	n/a	Triangular face, frontal bossing, up-slanting palpebral fissures, bulbous nasal tip	Triangular face, frontal bossing, up-slanting palpebral fissures, bulbous nasal tip	n/a	n/a	–
Limb contractures	+	n/a	+	+	+ (Distal)	n/a	+
Cardiorespiratory problems	Tachycardia, incomplete RBBB, respiratory failure	n/a	PDA (closed) and ASD, respiratory failure, bronchomalacia of right upper lobe bronchus	ASD (small), respiratory failure	Patent foramen ovale, respiratory failure	–	Severe pulmonary hypertension
Other findings and abnormalities	–	n/a	Bilateral undescended testicles, mild dilatation of the pelvis of the right kidney	Bilateral undescended testicles, mild hydronephrosis on the right side	Undescended testis	–	Thymus hyperplasia

*ASD*, autism spectrum disorder; *EEG*, electroencephalogram; *F*, female; *IUGR*, intrauterine growth restriction; *M*, male; *MRI*, magnetic resonance imaging; *n/a*, not available or not applicable; *PDA*, patent ductus arteriosus; *RBBB*, right bundle branch block.

To understand the function of *med11* in vivo, we generated a zebrafish model. To investigate the temporal and spatial expression pattern of *med11* messenger RNA (mRNA) during zebrafish development, we performed whole-mount RNA in situ hybridization. *Med11* mRNA is ubiquitously expressed at early stage of development by 24 hours after fertilization and more concentrated in head region as well as in lateral line primordium (Supplemental Figures 1A and 4A). By 5 dpf, *med11* mRNA was more restricted to sensory lateral line system (Supplemental Figures 1B and 4B); this is in line with the single cell RNA sequencing data from lateral line neuromast that showed *med11* expressed both in support and hair cells of the sensory lateral line neuromasts (Supplemental Figures 1C and 4C). Single cell RNA sequencing data from mouse showed *Med11* to be expressed in cochlea. Interestingly, 3 individuals affected by *med11* pathogenic variants exhibited bilateral hearing loss suggesting an important role of *med11* in hearing. We generated knock out animals (F0) using clustered regularly interspaced short palindromic repeats/Cas9 approach and analyzed the phenotypes in injected animals (F0 generation). The F0 knockout animals did not show any morphologic abnormalities by 3 dpf, however, at 4 dpf, small eyes, small brain (microcephaly), and heart edema (Figure 1O and P) was observed. About 50 % animals died by 10 dpf and remaining by 14 dpf (Figure 1Q). The zebrafish model recapitulated key clinical phenotypes exhibited by the affected individuals such as microcephaly, visual abnormalities, premature death, and cardiorespiratory phenotypes. Some *MED11* affected individuals showed exaggerated startle and bilateral hearing loss, therefore, we performed auditory evoked behavior response, and visual startle response to identify their response after startling the knockout animals with sound and light. *Med11* knockout animals showed reduced response compared with controls after the sound stimulus and showed increased movement after light stimulus suggesting compromised hearing and brain function (Figure 1R and S).

## Discussion

The *MED11* gene encodes for a subunit of the human MED complex, located in the head module and composed of 117 amino acids. The role of the complex on transcriptional activation and regulation is well-established.^1^^,^^2^ In this article, we reported 7 individuals from 5 different families affected with a novel autosomal recessive neurodegenerative disorder. The cardinal features of this disorder are congenital microcephaly, profound neurodevelopmental impairment, refractory myoclonic seizures, movement disorder with exaggerated startle responses, abnormal vision and hearing (ie, optic atrophy, sensorineural hearing impairment), severely progressive widespread CNS atrophy/degeneration with onset in prenatal age, and premature death occurring in early infancy or childhood.

Because the same recurrent p.Arg109Ter variant was identified in multiple unrelated families from the same geographical area (the Mediterranean basin), we looked specifically at whether there was a founder effect (ie, a single shared haplotype). However, haplotype analysis did not reveal evidence of a common ancestral haplotype across the investigated families, indicating that the variant had most likely occurred independently. Only 14 heterozygotes for the *MED11* p.Arg109Ter variant were identified in a total of more than half a million individuals across multiple population variant databases, indicating that the variant occurred in the homozygous state as an ultrarare mutational event. In addition, in the Genome Aggregation Database data set, there are 56 individuals carrying heterozygous loss-of-function truncating variants in *MED11* and none of these variants are present as homozygous, providing supportive evidence of pathogenicity for biallelic *MED11* variants resulting in changes of the gene reading frame. Notably, the homozygous *MED11* p.Arg109Ter variant leads to a premature truncation of the protein at amino acid position 109 and the loss of the last 9 conserved residues. The result is a putative MED11 product of 108 amino acids, with a resulting function that is highly likely to be impaired because of the disruption of the downstream C-terminal. This is also consistent with our RT-PCR and western blotting studies, which showed transcript and protein expression levels similar in mutant cell lines and wild-type controls. Interestingly, previous studies identified a direct interaction between MED11 and MED22, involving the formation of a 4-helix bundle domain with the C-terminal extensions of these 2 proteins also binding to the head subunit MED17.^1^^,^^9^ Furthermore, our simulations suggested that the disruption of MED11 C-terminal may affect the interactions among the 3 helices of the MED11, MED28, and MED30C subunits. Thus, it is possible that the deletion of the last 9 conserved intersubunit residues could destabilize the MED-complex dynamics and compromise its function; alternatively, a downstream binding site for a transcription factor within the C-terminal could also be altered as a result of the variant.

This work adds a further line of evidence that brain developmental and degenerative disorders may result from rare biallelic genetic defects of MED subunits (Supplemental Figures 1 and 2). Individuals carrying biallelic pathogenic variants in these genes (ie, *MED17*, *MED20*, *MED27*) share similar clinical phenotypes, variably characterized by global developmental delay, seizures (often refractory), congenital or postnatal microcephaly, recurrent hearing and/or vision abnormalities, and CNS atrophy/degeneration, particularly affecting the cerebellum and the brainstem. An overview of the phenotypes associated with deficits in different MED-complex subunits is available in Supplemental Figure 3 and Supplemental Table 2. Notably, no biallelic loss-of-function variants have been identified before this study in *MED17*, *MED20*, or *MED27*, suggesting how the genetic loss of MED-complex subunits may be not compatible with life or, alternatively, lead to very severe disruption in brain development and risk of (prenatal or early postnatal) lethality. Consistently, homozygous *MED11* knockout mouse mutants generated by the International Mouse Phenotyping Consortium showed either embryonic or preweaning lethality. Therefore, we performed functional studies in zebrafish because they grow externally, allowing us to follow the embryonic development. Zebrafish knockout animals recapitulated key clinical phenotypes, and die by 14 days of development, given all larval organs develop at this stage, it provides an opportunity to understand the pathophysiology of *med11*.

In summary, our results established *MED11* as a novel gene causing a severe neurodegenerative disorder potentially associated with premature death in humans. However, additional functional studies are needed to address the biological role of the MED11 subunit in both prenatal and postnatal brain physiology. Future studies on animal models and induced pluripotent stem cell-derived human brain organoids will be pivotal to understand the effect of the MED11 C-terminal conserved residues upon the overall MED-complex function and its downstream effects on the transcriptional machinery.

## Data Availability

The authors declare that the data supporting the findings of this study are available within the paper and its Supplemental Files.

## Conflict of Interest

G.Z. and C.B. are employers of CENTOGENE. All other authors declare no conflicts of interest.

## Additional Information

Individual A-II-1

Individual C-II-1
