# Supplementary material for: A homozygous MED11 C-terminal variant causes a lethal neurodegenerative disease
Source: Genet Med. 2022 Oct;24(10):2194–203. doi: 10.1016/j.gim.2022.07.013 (PMC10519206; doi:10.1016/j.gim.2022.07.013)
Supplement: Supplemental Table 2 [file mmc3.pdf]

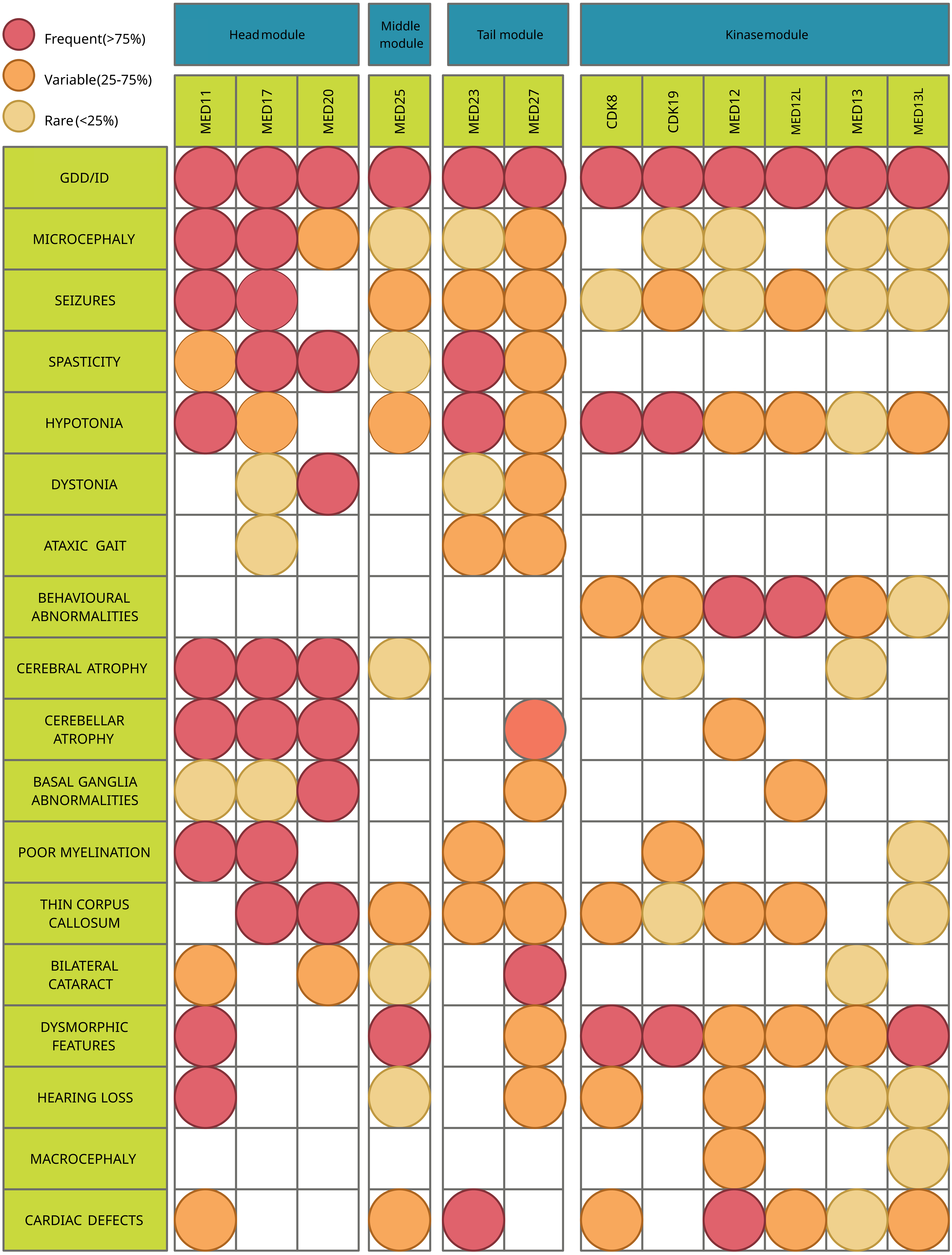
Frequent(>75%)
Variable(25-75%)
Rare (<25%)
Head module
Middle module
Tail module
Kinase module
MED11
MED17
MED20
MED25
MED23
MED27
CDK8
CDK19
MED12
MED12L
MED13
MED13L
GDD/ID
MICROCEPHALY
SEIZURES
SPASTICITY
HYPOTONIA
DYSTONIA
ATAXIC GAIT
BEHAVIOURAL ABNORMALITIES
CEREBRAL ATROPHY
CEREBELLAR ATROPHY
BASAL GANGLIA ABNORMALITIES
POOR MYELINATION
THIN CORPUS CALLOSUM
BILATERAL CATARACT
DYSMORPHIC FEATURES
HEARING LOSS
MACROCEPHALY
CARDIAC DEFECTS
