# Supplementary material for: A homozygous MED11 C-terminal variant causes a lethal neurodegenerative disease
Source: Genet Med. 2022 Oct;24(10):2194–203. doi: 10.1016/j.gim.2022.07.013 (PMC10519206; doi:10.1016/j.gim.2022.07.013)
Supplement: Supplementary data [file mmc4.docx]

Supplementary Table 1 Variant table

|  | MED11 |
| --- | --- |
| GRCh38/hg38 DNA Change | NC_000017.11 g.4733158C>T |
| cDNA/Coding Sequence Change [NM_001001683.2] | c.325C>T |
| Protein Change [NP_001001683.1] | p.Arg109Ter |
| Exon or Intron (Number Position) [GRCh38/hg38] | exon 3 of 3 position 109 of 558 (coding) |
| Codon Change | Cga/Tga |
| Variant Type | stop_gained |
| Zygosity | Homozygous |
| dbSNP ID | rs770295047 |
| Affected Individuals | 7 |
| gnomAD v3.1.1 (Highest Subpopulation) | 0.000006573 (East Asian 0.0001925) |
| gnomAD v2.1.1 (Highest Subpopulation) | 0.00003581 (East Asian 0.0003806) |
| Ensembl Browser | 0.0001217 (NCBI alfa) |
| Iranome | Absent |
| Centogene | 0.00001176 |
| GME Variome | Absent |
| UK Biobank | 0.0000056 |
| In-house Database | Absent |
| King Faisal Specialist Hospital and Research Center | Absent |
| GERP | 1.25 |
| CADD_Phred | 38 |
| Polyphen-2 | - |
| SIFT | - |
| Provean | - |
| MutationTaster | Disease causing (0.9999) |
| Clinical significance | Uncertain significance/ Likely pathogenic (PS4, PM2, PM4, PP3) |

**Supplementary Methods.**

**Homozygosity mapping and haplotype analysis**

Homozygosity mapping and haplotype analysis were performed on three affected individuals, two parents and five unrelated healthy controls from the same ethnic background. A region of 2.5Mb upstream and downstream the variant of interest was extracted from gVCF files using vcftools. GATK 4.1.4.0 was used to combine the extracted region across the different gVCF files and perform joint genotyping. The resulting file was normalised using bcftools norm. Homozygosity mapping was carried out by loading the output file onto homozygosity mapper (http://homozygositymapper.org) and the region of interest was visually inspected. After filtering out low-quality variants from the same file, the haplotype region was analysed in excel.

**Western blotting**

Western blot analysis was performed extracting protein and RNA from fibroblasts derived from Individual A.II.1 and his unaffected mother Control proteins were extracted from control fibroblast cell lines from healthy, unrelated individuals (3 years old and 24 years old). Cells were lysed in radioimmunoprecipitation assay buffer [150 mM NaCl, 1.0% IGEPAL® CA-630, 0.5% sodium deoxycholate, 0.1% SDS, 50 mM Tris (Sigma)], to which Protease Inhibitor Tablets were added to avoid proteolytic degradation (Pierce Protease Inhibitor Tablets, Thermoscientific), for 1 hour in ice, vortexing intermittently, followed by centrifugation at 14,600 rpm for 10-15 minutes at 4° C to retrieve the supernatants. Total protein concentrations were determined by Bicinchoninic acid protein assay (PierceTM). Protein lysates were diluted 1:1 with Nu PAGE LDS sample buffer, and resolved by % PAGE, transferred onto nitrocellulose membrane and incubated in blocking solution [ Phosphate-Buffered Saline (PBS), 3% ECL blocking agent, 0.1 % Tween-20] for 1 h at room temperature, followed by overnight incubation with primary antibody at 4 C [MED11, Abcam, 1:700 dilution; Anti-Actin, Sigma,1 1:5000 dilution ]. Membranes were washed with PBS-T [Phosphate-Buffered Saline, 0.1% Tween-20] five times for five minutes followed by incubation with secondary antibody (HRP conjugated anti-rabbit or anti-mouse) for 1 h at room temperature. Blots were visualized by chemiluminescence using the Amersham ECL Prime Substrate [ ECL Prime Luminol Enhancer Solution, ECL Primer Peroxide Solution, 1:1 dilution]. Data were analysed using actin as housekeeping protein. The experiment was repeated three times.

**Semi-quantitative RT-PCR**

Total RNA was extracted (RNeasy® Mini kit, Qiagen) according to the manufacturer’s protocol, from cultured primary fibroblasts obtained from Individual A-II-1, his mother and two healthy controls. RNA samples (1 μg) were reverse transcribed with SuperScript First-Strand Synthesis system and randomer hexamers as primers (Life Technologies, Carslbad, CA, USA). The expression levels of MED11 were measured on RNA using the following primers: MED11_Fw: CAGAATGCAGGTACTGTGAT , MED11_Rv: GCAGCATGAAGTGTTTGAGG ; GAPDH Fw GAGTCAACGG: , GAPDH_Rv TTGATTTTGGAGGATCTCG: . Non-RT RNA sample was used to control for genomic DNA contamination. PCR reactions were optimized for the gene-specific primer. Amplified products were resolved on 2% agarose gels and visualized. Data were analyzed using glyceraldeide 3-phosphate dehydrogenase (GAPDH) as the housekeeping gene. The experiment was repeated three times.

**Computational studies.**

The mediator complex was built by homology by using the cryo-EM structure as a template^1^. Missing residues were included in the structure of head module and proximal tail by homology modelling by using SWISS-MODEL webserver^2^ and in particular, med8, med17, med18 and med20 structures were refined. Then, the subunits closer to med11 in the head module and the proximal tail were isolated to perform the molecular dynamics (MD) simulations, namely: med8, med11, med17, med18, med20, med27, med28, med30C. Region 109-116 of med11 was removed to create the nonsense mutation at R109. Thus, two systems containing the WT med11 and the R109 nonsense mutant underwent the following MD protocol. Each model was placed in a cubic box with a water layer of 1.0nm, neutralised with Na+ and/or Cl- ions, and minimised. The steepest descent minimization stopped either when the maximum force was lower than 1000.0 kJ/mol/nm or when 50000 minimisation steps were performed with 0.005 kJ/mol energy step size, Verlet cutoff scheme, short-range electrostatic cut-off and Van der Waals cut-off of 1.0nm. AMBER99SB-ILDN force field^3^, tip3p water, and periodic boundary conditions were employed. NVT and NPT equilibrations were performed for 100 ps by restraining the protein backbone, followed by 500 ns long NPT production runs at 330 K. The iteration time step was set to 2 fs with the Verlet integrator and LINCS^4^ constraint. All the simulations and their analysis were run as implemented in the Gromacs package v. 2020.3^5^. RMSD values of med28 and med30C residues that interact with med11 C-terminal were calculated from trajectory configurations sampled every 0.5ns by previously fitting the med11 subunit with respect to its structure at t=0. We clustered 600 mutant mediator complex poses obtained from the trajectory after 200 ns by using the Daura method^6^, as implemented in Gromacs, with a cut-off of 0.1 nm. We employed the med28 and med30C residues that interact with med11 C-terminal for the fit and RMSD matrix calculation in the clustering. The the most representative complex conformation was chosen as the representative structure of the biggest cluster, which contained 95% of structures, and was the one obtained at 310.5 ns in the trajectory. Simulations were run on M100 (CINECA, Italy).

1. Srinivasan Rengachari, Sandra Schilbach, Shintaro Aibara, Christian Dienemann, Patrick Cramer, Structure of human Mediator-RNA polymerase II transcription pre-initiation complex. bioRxiv 2021.03.11.435010; doi: https://doi.org/10.1101/2021.03.11.435010.

2. Waterhouse, A., Bertoni, M., Bienert, S., Studer, G., Tauriello, G., Gumienny, R., Heer, F.T., de Beer, T.A.P., Rempfer, C., Bordoli, L., Lepore, R., Schwede, T. SWISS-MODEL: homology modelling of protein structures and complexes. Nucleic Acids Res. 46, W296-W303 (2018).

3. Lindorff-Larsen, K.; Piana, S.; Palmo, K.; Maragakis, P.; Klepeis, J.L.; Dror, R.O.; Shaw, D.E. Improved side-chain torsion potentials for the Amber ff99SB protein force field. Proteins 2010, 78, 1950–1958.

4. Hess, B.; Bekker, H.; Berendsen, H.J.C.; Fraaije, J. LINCS: A linear constraint solver for molecular simulations. J. Comput. Chem. 1997, 18, 1463–1472.

5. M.J. Abraham, T. Murtola, R. Schulz, S. Páll, J.C. Smith, B. Hess, and E. Lindahl, “GROMACS: High performance molecular simulations through multi-level parallelism from laptops to supercomputers,” SoftwareX, 1–2 19–25 (2015).

6. Daura, X.; van Gunsteren, W.F.; Jaun, B.; Mark, A.E.; Gademann, K.; Seebach, D. Peptide folding: When simulation meets experiment. Angew. Chem. Int. Ed. 1999, 38, 236–240.

**Supplementary Figure 1.
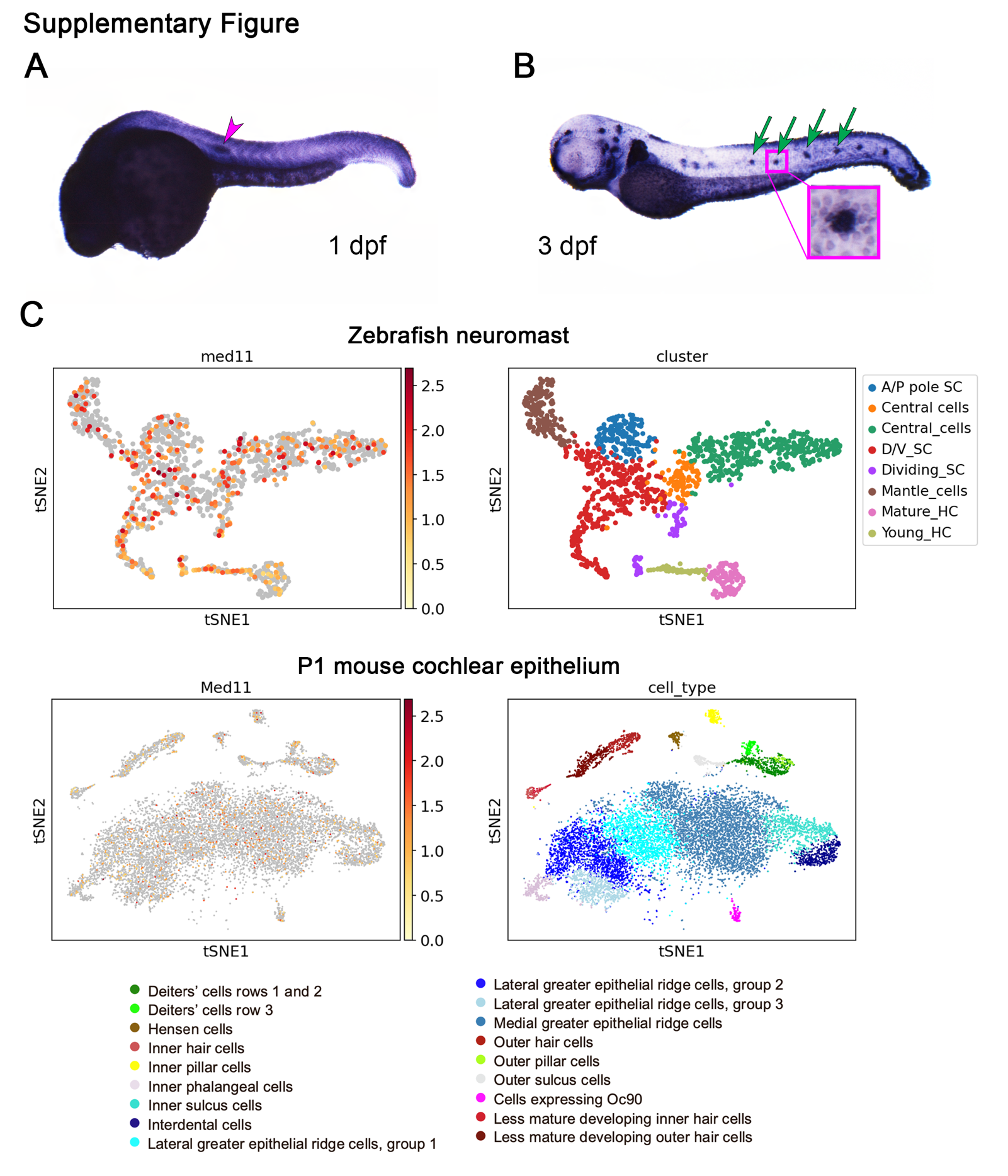
**

**Supplementary Figure 1.** mRNA expression of Med11 in zebrafish and mouse. A) Whole mount mRNA in situ hybridization showing mRNA expressed ubiquitously in zebrafish embryos at early stage, and more prominent in certain organs and tissues such as brain and lateral line primordium (violet arrowhead). B) By 3 days post fertilization (3 dpf), med11 mRNA is highly restricted to sensory lateral line neuromasts (green arrow and violet square).  C) tSNE plot of *med11* mRNA expression from single-Cell RNAseq data also confirmed *med11* RNA enriched both in support and hair cells of the lateral line neuromasts. D) Single Cell RNA seq on mouse cochlea showed *Med11* mRNA expression in mouse cochlea


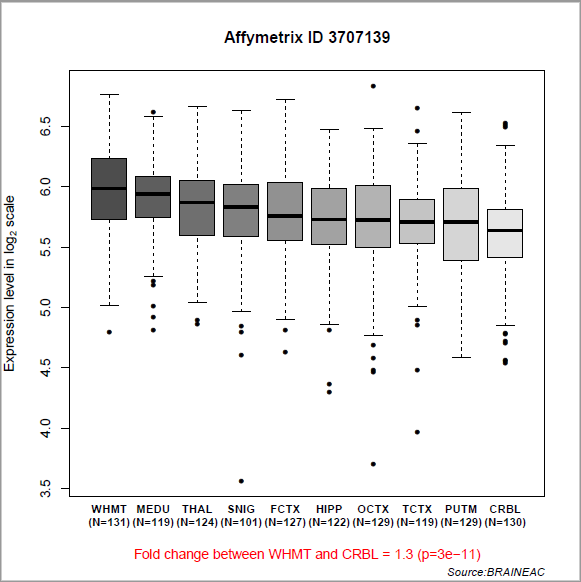


**Supplementary Figure 2.**
MED11 gene expression across ten brain tissues: cerebellar cortex (CRBL), frontal cortex (FCTX), hippocampus (HIPP), medulla (specifically inferior olivary nucleus, MEDU), occipital cortex (specifically primary visual cortex, OCTX), putamen (PUTM), substantia nigra (SNIG), thalamus (THAL), temporal cortex (TCTX) and intralobular white matter (WHMT).


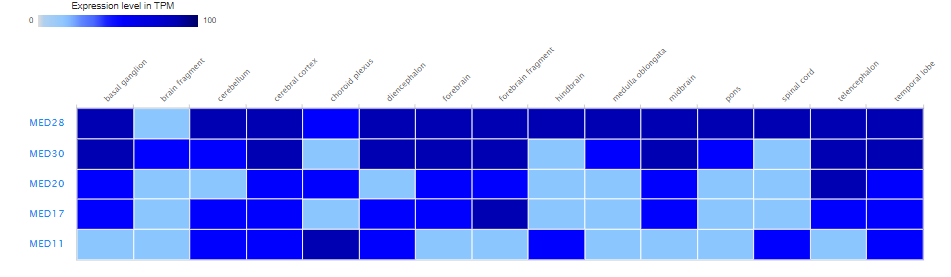


**Supplementary Figure 3.**

Head module gene expression in prenatal human brain development (Developmental stage: 9 post conceptional weeks). License <http://www.apache.org/licenses/LICENSE-2.0>

**Supplementary Figure 4.** Human Mediator Complex and MEDopathies.
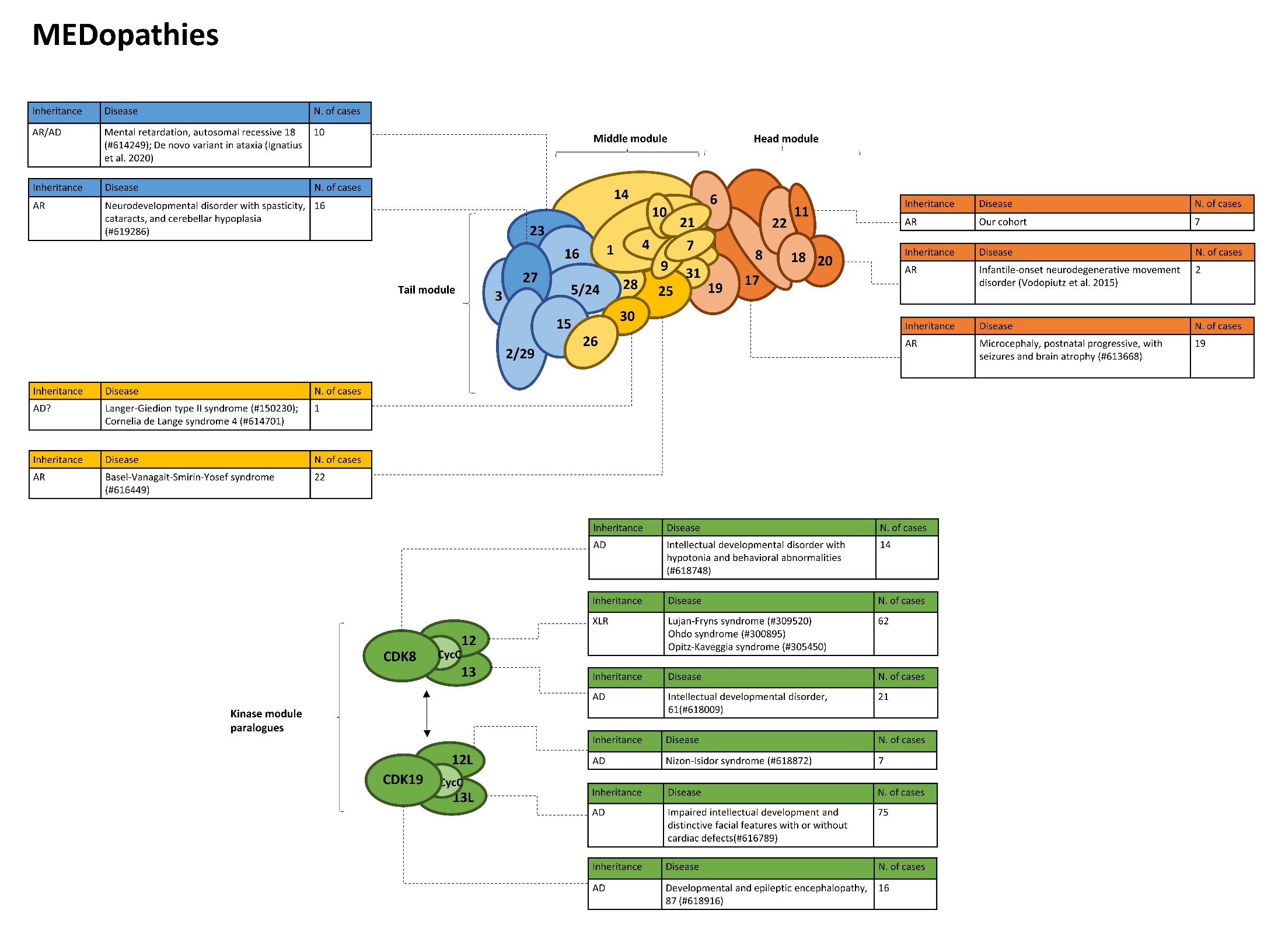


**SYNAPSE Study Group.**

| **Collaborators sharing data or patients for genetic analysis for comparative and variant analysis** | **Affiliations of SYNAPS Study Group Collaborators** |
| --- | --- |
| Prof Michael G Hannah | Affiliation: Department of Neuromuscular Diseases, Queen Square Centre for Neuromuscular Diseases, UCL Queen Square Institute of Neurology and National Hospital for Neurology and Neurosurgery, London WC1N 3BG, UK. |
| Dr. Enrico Bugiardini | Affiliation: Department of Neuromuscular Disease, UCL Queen Square Institute of Neurology and The National Hospital for Neurology, London, UK. |
| Prof Yamna Kriouile | Affiliation: Unit of Neuropediatrics, Children's Hospital of Rabat, University of Rabat, Rabat 6527, Morocco  Email: [d](mailto:barakatamina@hotmail.fr)[r.kriouile@gmail.com](mailto:r.kriouile@gmail.com) |
| Prof. Mohamed El Khorassani | Affiliation: Children's Hospital of Rabat, University of Rabat, Rabat 6527, Morocco  Email: [elkhorassani.mohamed@gmail.com](mailto:elkhorassani.mohamed@gmail.com) |
| Prof. Mhammed Aguennouz | Affiliation: Department of Clinical and Experimental Medicine, University of Messina, Messina 98123, Italy  Email: [aguenoz@unime.it](mailto:aguenoz@unime.it) |
| Prof Stanislav Groppa | Affiliation: Department of Neurology and Neurosurgery, Institute of Emergency Medicine, Chisinau, Republic of Moldova.  Email: [sgroppa@gmail.com](mailto:sgroppa@gmail.com) |
| Dr. Blagovesta Marinova Karashova | Affiliation: Department of Paediatrics, Medical University of Sofia, Sofia 1431, Bulgaria Email: [blagovestakarashova@gmail.com](mailto:blagovestakarashova@gmail.com) |
| Dr Gabriella Di Rosa, MD, PhD | Affiliation: Department of Pediatrics, University of Messina, Messina 98123, Italy  Email: gdirosa@unime.it |
| Prof. Jatinder S. Goraya, MD, FRCP | Affiliation: Division of Paediatric Neurology, Dayanand Medical College & Hospital, Ludhiana, Punjab 141001, India  Email: gorayajs@gmail.com |
| Prof. Tipu Sultan | Affiliation: Division of Paediatric Neurology, Children's Hospital of Lahore, Lahore 381-D/2, Pakistan  Email: [tipusultanmalik@hotmail.com](mailto:tipusultanmalik@hotmail.com) |
| Prof. Daniela Avdjieva, | Affiliation: Department of Paediatrics, Medical University of Sofia, Sofia 1431, Bulgaria  Email: [davdjieva@yahoo.com](mailto:davdjieva@yahoo.com) |
| Dr. Hadil Kathom, | Affiliation: Department of Pediatrics, Medical University of Sofia, Sofia 1431, Bulgaria  Email: [hadilmk@gmail.com](mailto:hadilmk@gmail.com) |
| Prof. Dr Radka Tincheva | Affiliation: Head of Department of Clinical Genetics, University Pediatric Hospital, Sofia 1431, Bulgaria  Email: [radka.tincheva@gmail.com](mailto:radka.tincheva@gmail.com) |
| Prof. Selina Banu | Affiliation: Neurosciences Unit, Institute of Child Health and Shishu Shastho Foundation Hospital, Mirpur, Dhaka 1216, Bangladesh  Email: [selinabanu17@gmail.com](mailto:selinabanu17@gmail.com) |
| Prof. Pierangelo Veggiotti | Affiliation: Unit of Infantile Neuropsychiatry Fondazione  Istituto Neurologico "C. Mondino" IRCCS, Via Mondino 2, Pavia 27100, Italy  Email: [pierangelo.veggiotti@unipv.it](mailto:pierangelo.veggiotti@unipv.it) |
| Prof. Alberto Verrotti | Affiliation: University of L’Aquila, L’Aquila, Italy  Email: [verrottidipianella@univaq.it](mailto:verrottidipianella@univaq.it) |
| Dr. Salvatore Savasta | Affiliation: Division of Pediatric Neurology, Department of Pediatrics, University of Pavia, IRCCS Policlinico "San Matteo", Pavia 27100, Italy  Email: [S.Savasta@smatteo.pv.it](mailto:S.Savasta@smatteo.pv.it) |
| Dr. Elisa Cali | Affiliation: Department of Neuromuscular Disease, UCL Queen Square Institute of Neurology and The National Hospital for Neurology, London, UK. |
| Dr Reza Maroofian | Affiliation: Department of Neuromuscular Disease, UCL Queen Square Institute of Neurology and The National Hospital for Neurology, London, UK. |
| Dr. Alfons Macaya Ruiz | Affiliation: University Hospital Vall d'Hebron, Barcelona 08035, Spain  Email: amacaya@vhebron.net |
| Prof. Barbara Garavaglia | Affiliation: IRCCS Foundation, Neurological Institute “Carlo Besta”, Molecular Neurogenetics, 20126 Milan, Italy  Email: segr.neurogenetica@istituto-besta.it |
| Dr. Eugenia Borgione | Affiliation: Laboratorio di Neuropatologia Clinica, U.O.S. Malattie, Neuromuscolari Associazione OASI Maria SS. ONLUS – IRCCS, Via Conte Ruggero 73, 94018 Troina, Italy  Email: eborgione@oasi.en.it |
| Prof. Savvas Papacostas | Affiliation: Neurology Clinic B, The Cyprus Institute of Neurology and Genetics, 6 International Airport Road, 1683 Nicosia, Cyprus  Email: savvas@cing.ac.cy |
| Dr. Chiara Compagnoni | Affiliation: Department of Biotechnological and Applied Clinical Sciences, University of L’Aquila, 67100, Italy |
| Dr. Alessandra Piccirilli | Affiliation: Department of Biotechnological and Applied Clinical Sciences, University of L’Aquila, 67100, Italy |
| Dr. Michail Vikelis | Affiliation: Iatreio Kefalalgias Glyfadas, 8 Lazaraki str, 3rd floor, 16675, Athens, Greece  Email: [mvikelis@headaches.gr](mailto:mvikelis@headaches.gr) |
| Prof Henry Houlden | Affiliation: Department of Molecular Neuroscience, University College London, London, UK  Email: [h.houlden@ucl.ac.uk](mailto:h.houlden@ucl.ac.uk) |
| Dr Viorica Chelban | Affiliation: Department of Molecular Neuroscience, University College London, London, UK  Email: v.chelban@ucl.ac.uk |
| Prof. Vincenzo Salpietro | Affiliation: Department of Molecular Neuroscience, University College London, London, UK  Email: [v.salpietro@ucl.ac.uk](mailto:v.salpietro@ucl.ac.uk)  Chair of Paediatrics, University of L’Aquila, Italy |
| Dr. Stephanie Efthymiou | Affiliation: Department of Molecular Neuroscience, University College London, London, UK  Email: s.efthymiou@aucl.ac.uk |
| Dr Rauan Kaiyrzhanov | Affiliation: Department of Molecular Neuroscience, University College London, London, UK  Email: rauan.kaiyrzhanov.14@ucl.ac.uk |
| Prof. Andrea Cortese | Department of Neuromuscular Diseases, University College London, London, United Kingdom  Department of Brain and Behavioral Sciences, University of Pavia, Pavia, Italy |
| Dr. Roisin Sullivan | Affiliation: Department of Molecular Neuroscience, Queen's Square Institute of Neurology, UCL, London, UK.  [r.sullivan@ucl.ac.uk](mailto:r.sullivan@ucl.ac.uk) |
| Prof Eleni Zamba Papanicolaou | Affiliation: The Cyprus Institute of Neurology & Genetics, Nicosia, Cyprus  Email: [ezamba@cing.ac.cy](mailto:ezamba@cing.ac.cy) |
| Dr Efthymios Dardiotis | Affiliation: UNIVERSITY HOSPITAL OF LARISSA, NEUROLOGY Department, Greece  Email: [edar@med.uth.gr](mailto:edar@med.uth.gr) |
| Prof Shazia Maqbool | Affiliation: Department of Developmental and Behavioral Pediatrics, CH&ICH, Lahore, Pakistan  Email: [drshazimaq@yahoo.com](mailto:drshazimaq@yahoo.com) |
| Prof Shahnaz Ibrahim | Affiliation: Department of Pediatrics and child health, Aga Khan University, Karachi, Pakistan  Email: [shahnaz.ibrahim@aku.edu](mailto:shahnaz.ibrahim@aku.edu) |
| Prof Salman Kirmani | Affiliation: Department of Paediatrics & Child Health, The Aga Khan University, Karachi , Pakistan  Email: [salman.kirmani@aku.edu](mailto:salman.kirmani@aku.edu) |
| Dr. Nuzhat Noureen Rana | Affiliation: Department of Paediatric Neurology, Children Hospital Complex and ICH, Multan, Pakistan  Email: [drnuzhatrana@gmail.com](mailto:drnuzhatrana@gmail.com) |
| Dr. Osama Atawneh | Affiliation: Hilal Pediatric Hospital Hebron, Hebron West Bank, Palestine  Email: [osamaat@gmail.com](mailto:osamaat@gmail.com) |
| Prof Shen-Yang Lim | Affiliation: Department of Biomedical Science, Faculty of Medicine, University of Malaya, Malaysia  Email: [limshenyang@gmail.com](mailto:limshenyang@gmail.com) |
| Dr Farooq Shaikh | Affiliation: Jeffrey Cheah School of Medicine and Health Sciences, Monash University Malaysia  Email: [farooq.shaikh@monash.edu](mailto:farooq.shaikh@monash.edu) |
| Annarita Scardamaglia | Department of Neuromuscular Diseases, University College London, London, United Kingdom  [a.scardamaglia@ucl.ac.uk](mailto:a.scardamaglia@ucl.ac.uk) |
| Prof George Koutsis | Affiliation: Neurogenetics Unit, Neurology Department, Eginition Hospital, National and Kapodistrian University, Athens, Greece  Email: [marianthibr@med.uoa.gr](mailto:marianthibr@med.uoa.gr) |
| Prof Salvatore Mangano | Affiliation: Unità di Neuropsichiatria Infantile, AOUP "P.Giaccone" Palermo, Italy  Email: [salvatore.mangano@unipa.it](mailto:salvatore.mangano@unipa.it) |
| Dr Carmela Scuderi | Affiliation: Associazione Oasi Maria SS, 94018 Troina, Italy  Email: [cscuderi@oasi.en.it](mailto:cscuderi@oasi.en.it) |
| Dr Eugenia Borgione | Affiliation: Associazione Oasi Maria SS, 94018 Troina, Italy  Email: [eborgione@oasi.en.it](mailto:eborgione@oasi.en.it) |
| Dr Giovanna Morello | Affiliation: Institute of Neurological Sciences, National Research Council, Mangone, Italy  Email: [g.morello@isn.cnr.it](mailto:g.morello@isn.cnr.it) |
| Prof Massimo Zollo | Affiliation: CEINGE, Biotecnologie Avanzate S.c.a.rl., Naples, Italy  Email: [massimo.zollo@unina.it](mailto:massimo.zollo@unina.it) |
| Dr Gali Heimer | Affiliation: University Hospital of Tel Aviv, Tel Aviv, Israel  Email: [galih.md@gmail.com](mailto:galih.md@gmail.com) |
| Prof Pasquale Striano | Affiliation: Institute “Giannina Gaslini”, Genova, Italy  Email: [strianop@gmail.com](mailto:strianop@gmail.com) |
| Dr Issam Al-Khawaja | Affiliation: Albashir University Hospital, Amman, Jordan  Email: [isamkhawaja61@gmail.com](mailto:isamkhawaja61@gmail.com) |
| Dr Fuad Al-Mutairi | Affiliation: King Saud University, Riyadh, Saudi Arabia  Email: [almutairifu@NGHA.MED.SA](mailto:almutairifu@NGHA.MED.SA) |
| Prof Fowzan S Alkuraya | Affiliation: King Faisal Specialist Hospital and Research Center, Riyadh, Saudi Arabia  Email: [falkuraya@kfshrc.edu.sa](mailto:falkuraya@kfshrc.edu.sa) |
| Dr Mie Rizig | Affiliation: Department of neuromuscular diseases, Queen square-Institute of neurology, University College London  Email: [mie.rizig@uclmail.net](mailto:mie.rizig@uclmail.net) |
| Dr. Chingiz Shashkin | Affiliation: Kazakh National State University, Almaty, Kazakhstan  Email: [chingizshashkin@gmail.com](mailto:chingizshashkin@gmail.com) |
| Dr. Nazira Zharkynbekova, | Affiliation: Shymkent Medical Academy, Kazakhstan  Email: [nazirazhar@mail.ru](mailto:nazirazhar@mail.ru) |
| Dr. Kairgali Koneyev, | Affiliation: Kazakh National State University, Almaty, Kazakhstan  Email: [kairgali@mail.ru](mailto:kairgali@mail.ru) |
| Dr. Ganieva Manizha, | Affiliation: Avicenna Tajik State Medical University, Dushanbe, Tajikistan  Email: [ganieva.manizha.79@mail.ru](mailto:ganieva.manizha.79@mail.ru) |
| Dr. Maksud Isrofilov | Affiliation: Avicenna Tajik State Medical University, Dushanbe, Tajikistan  Email:dr.maks-55@mail.ru |
| Dr. Ulviyya Guliyeva, | Affiliation: Mediclub clinic, Baku, Azerbaijan  Email: [doctor.ulya@gmail.com](mailto:doctor.ulya@gmail.com) |
| Dr. Kamran Salayev | Affiliation: Azerbaijan State Medical University, Baku, Azerbaijan  Email: [ksalayev@yahoo.com](mailto:ksalayev@yahoo.com) |
| Dr. Samson Khachatryan | Affiliation: "Somnus" Neurology Clinic Sleep and Movement Disorders Center, Yerevan, Armenia.  Email: [drsamkhach@gmail.com](mailto:drsamkhach@gmail.com) |
| Dr. Georgia Xiromerisiou | Affiliation: Department of Neurology, Medical School, University of Thessaly, Larissa, Greece  Email: [geoksirom@med.uth.gr](mailto:geoksirom@med.uth.gr), [georgiaxiromerisiou@gmail.com](mailto:georgiaxiromerisiou@gmail.com) |
| Dr. Cleanthe Spanaki | Affiliation: Department of Neurology, Medical School, University of Crete, Heraklion, Greece  Email: [kliospanaki@med.uoc.gr](mailto:kliospanaki@med.uoc.gr) |
| Dr Arianna Tucci | Affiliation: William Harvey Research Institute, The NIHR Biomedical Research Centre at Barts, Queen Mary University London, London, UK  Email: a.tucci@qmul.ac.uk |
| Dr. Chiara Fiorillo | Affiliation: Department of Neuroscience, Rehabilitation, Ophthalmology, Genetics, Maternal and Child Health, University of Genova, Genoa, Italy. |
| Dr. Federico Rissotto | Affiliation: Department of Ophthalmology, IRCCS San Raffaele Scientific Institute, Milan, Italy |
| Dr. Francina Munell | Affiliation: Pediatric Department, Vall d'Hebron Hospital, Barcelona, Spain. |
| Dr. Antonella Gagliano | Affiliation: Department of Biomedical Sciences, Section Neuroscience and Clinical Pharmacology, University of Cagliari, Cagliari, Italy; Child and Adolescent Neuropsychiatry, "A. Cao'' Paediatric Hospital, "G. Brotzu" Hospital Trust, Via E. Jenner, 09121 Cagliari, Italy. |
| Dr. Farida Jan | Affiliation: Department of Paediatrics and Child Health, Aga Khan University Hospital, Karachi, Pakistan |
| Prof. Roberto Chimenz | Affiliation: Unit of Pediatric Nephrology, and Rheumatology with Dialysis, Department of Human Pathology in Adult and Developmental Age "Gaetano Barresi", University of Messina, "G. Martino" Policlinic, 98124 Messina, Italy. |
| Prof. Eloisa Gitto | Affiliation: Department of Pediatrics, University of Messina, Messina, Italy. |
| Dr. Caterina Cuppari | Affiliation: Department of Pediatrics, University of Messina, Messina, Italy. Electronic address: katia.cuppari@libero.it. |
| Prof Carmelo Romeo | Affiliation: Department of Adult and Childhood Human Pathology, Unit of Pediatrics, University Hospital of Messina, Messina, Italy. |
| Dr. Francesca Magrinelli | Affiliation: Department of Clinical and Movement Neurosciences, UCL Queen Square Institute of Neurology, London, UK.  Department of Neurosciences, Biomedicine and Movement Sciences, University of Verona, Verona, Italy. |
| Prof. Neerja Gupta | Affiliation: Division of Genetics, Department of Pediatrics, All India Institute of Medical Sciences, New Delhi, 110029, India. neerja17@gmail.com. |
| Prof Madhulika Kabra | Affiliation: Division of Genetics, Department of Pediatrics, All India Institute of Medical Sciences, New Delhi, 110029, India. |
| Dr. Hanene Benrhouma | Affiliation: Child and Adolescent Neurology Department of Neurology, National Institute of Neurology, Tunis, Tunisia. |
| Dr. Meriem Tazir | Affiliation: Service de Neurologie, University Hospital Mustapha Bacha, Alger, Algeria. meriem.tazir@sante.dz |
| Dr. Luca Zagaroli | Affiliation: Department of Pediatrics, University of L’Aquila, 67100, Italy |
| Dr. Claudia Caloisi | Affiliation: Department of Pediatrics, University of L’Aquila, 67100, Italy |
| Dr. Cecilia Fabiano | Affiliation: Department of Pediatrics, University of L’Aquila, 67100, Italy |
| Dr. Gabriella Bottone | Affiliation: Department of Pediatrics, University of L’Aquila, 67100, Italy |
| Dr. Giovanni Farello | Affiliation: Department of Clinical Medicine, Public Health, Life and Environmental Sciences, University of L'Aquila, 67100 L'Aquila, Italy |
| Dr Sandra Di Fabio | Affiliation: Department of Pediatrics, University of L’Aquila, 67100, Italy |
| Prof. Makram Obeid | Affiliation: Division of Child Neurology, Department of Pediatric and Adolescent Medicine, American University of Beirut Medical Center, Beirut, Lebanon. mo21@aub.edu.lb. |
| Dr. Sophia Bakhtadze | Affiliation: Department of Paediatric Neurology, Tbilisi State Medical University, 0160, Tbilisi, Georgia. Electronic address: s.bakhtadze@tsmu.edu. |
| Prof. Nebal W Saadi | Affiliation: College of Medicine, Baghdad University, Baghdad, Iraq.  Children Welfare Teaching Hospital, Baghdad, Iraq. |
| Prof Maha S Zaki | Affiliation: Clinical Genetics Department, Human Genetics and Genome Research Division, National Research Centre, Cairo, Egypt |
| Prof. Chahnez C Triki | Affiliation: Department of Child Neurology, Hedi Chaker Hospital, LR19ES15 Sfax University, Sfax, Tunisia. |
| Dr. Majdi Kara | Affiliation: Department of Pediatrics, University of Tripoli, Tripoli, Libya. |
| Dr. Vincenzo Belcastro | Affiliation: Neurology Unit, Department of Medicine, S. Anna Hospital , Como , Italy. |
| Dr. Nicola Specchio | Affiliation: Rare and Complex Epilepsy Unit, Department on Neuroscience, Bambino Gesu' Children's Hospital, IRCCS, Member of the ERN EpiCARE, Rome, Italy |
| Dr. Ehsan G Karimiani | Affiliation: Genetics Research Centre, Molecular and Clinical Sciences Institute, St. George's, University of London, London, UK.  Next Generation Genetic Polyclinic, Razavi International Hospital, Mashhad, Iran. |
| Dr. Ahmed M Salih | Affiliation: Molecular microbiology & Immunology, College of Medicine, University of Duhok, Duhok, Kurdistan Region, Iraq. |
| Prof. Luca A Ramenghi | Affiliation: Neonatal Intensive Care Unit, Giannina Gaslini Children's Hospital, Genova, Italy. Electronic address: lucaramenghi@gaslini.org. |
| Dr. Emanuele David | Affiliation: Radiology Unit, Papardo-Hospital, Messina, Italy, Messina, Italy. |
| Dr. Riccardo Curró | Affiliation: Department of Neuromuscular Diseases, University College London, London, United Kingdom; Department of Brain and Behavioral Sciences, University of Pavia, Pavia, Italy |
| Dr. Maria Laura Iezzi | Affiliation: Department of Pediatrics, University of L’Aquila, 67100, Italy |
| Dr. Giulia Iapadre | Affiliation: Department of Pediatrics, University of L’Aquila, 67100, Italy |
| Dr. Giuliana Nanni | Affiliation: Department of Pediatrics, University of L’Aquila, 67100, Italy |
| Dr. Giovanna Scorrano | Affiliation: Department of Pediatrics, University of L’Aquila, 67100, Italy |
| Dr. Maria F. Fiorile | Affiliation: Department of Pediatrics, University of L’Aquila, 67100, Italy |
| Prof. Francesco Brancati | Affiliation: Department of Life, Health and Environmental Sciences, University of L'Aquila, 67100 L'Aquila, Italy;  Human Functional Genomics, IRCCS San Raffaele Roma, 00163 Rome, Italy. |
| Dr. Giovanna Di Falco | Affiliation: Consorzio Siciliano di Riabilitazione, Sicily, Italy |
| Dr. Luana Mandarà | Affiliation: Unit of Medical Genetics, Ragusa ASP, Ragusa, Italy |
| Dr. Giuseppe Barrano | Affiliation: Unit of Medical Genetics, Ragusa ASP, Ragusa, Italy |
| Dr. Maurizio Elia | Affiliation: Unit of Neurology and Clinical Neurophysiopathology, Oasi Institute for Research on Mental Retardation and Brain Aging (IRCCS), Troina, EN, Italy. |
| Dr. Gaetano Terrone | Affiliation: Department of Child Neurology and Psychiatry, University Federico II, Napoli, Italy. |
| Dr. Francesca F. Operto | Affiliation: Department of Pediatrics University of Salerno |
| Dr. Mariella Valenzise | Affiliation: Department of Pediatrics University of Messina |
| Dr. Ylenia Della Rocca | Affiliation: Department of Biotechnological and Applied Clinical Sciences, University of L’Aquila, 67100, Italy Department of Innovative Technologies in Medicine and in Dentistry, University “G. D’Annunzio”, Chieti-Pescara, Italy |
| Dr. Francesca Zazzeroni | Affiliation: Department of Biotechnological and Applied Clinical Sciences, University of L’Aquila, 67100, Italy |
| Dr. Edoardo Alesse | Affiliation: Department of Biotechnological and Applied Clinical Sciences, University of L’Aquila, 67100, Italy |
| Dr. Filippo Manti | Affiliation: Unit of Child Neurology and Psychiatry, Department of Human Neuroscience, Sapienza University of Rome, Rome, Italy. |
| Dr. Serena Galosi | Affiliation: Unit of Child Neurology and Psychiatry, Department of Human Neuroscience, Sapienza University of Rome, Rome, Italy. |
| Dr. Francesca Nardecchia | Affiliation: Unit of Child Neurology and Psychiatry, Department of Human Neuroscience, Sapienza University of Rome, Rome, Italy. |
| Prof. Vincenzo Leuzzi | Affiliation: Unit of Child Neurology and Psychiatry, Department of Human Neuroscience, Sapienza University of Rome, Rome, Italy. |
| Dr. Erica Pironti | Affiliation: Department of Human Pathology of the Adult and Affiliation: Developmental Age "Gaetano Barresi", Unit of Child Neurology and Psychiatry, University of Messina, Messina, Italy. |
| Dr. Greta Amore | Affiliation: Department of Human Pathology of the Adult and Developmental Age "Gaetano Barresi", Unit of Child Neurology and Psychiatry, University of Messina, Messina, Italy. |
| Dr. Giorgia Ceravolo | Affiliation: Department of Human Pathology of Adulthood and Childhood, Pediatric Emergency Unit, University of Messina, Messina, Italy  UCL Institute of Neurology, London WC1N 3BG, United Kingdom |
| Dr. Faisal Zafar | Affiliation: Children's Hospital Multan, Pakistan |
| Prof Ehsan Ullah | Affiliation: Children's Hospital Multan, Pakistan |
| Dr Erum Afzal | Affiliation: Children's Hospital Multan, Pakistan |
| Dr Iram Javed | Affiliation: Childrens Hospital Faisalabad, Pakistan |
| Dr Fatima Rahman | Childrens Hospital Lahore, Pakistan |
| Dr Muhammad Mehboob Ahmed | Childrens Hospital Faisalabad, Pakistan |
| Dr. Pasquale Parisi | Affiliation: Affiliation: Chair of Pediatrics, NESMOS Department, Faculty of Medicine and Psychology, Sapienza University of Rome, Sant'Andrea Hospital. |
| Dr. Paola Borgia | Affiliation: Department of Neuroscience, Rehabilitation, Ophthalmology, Genetics, Maternal and Child Health, University of Genova, Genoa, Italy. |
| Dr. Giuseppe D Mangano | Affiliation: Department of Health Promotion, Mother and Child Care, Internal Medicine and Medical Specialities G. D’Alessandro,” University of Palermo, Palermo, Italy. |
| Prof. Francesco Chiarelli | Department of Pediatrics, University of Chieti, Via dei Vestini 5, 66100 Chieti, Italy. |
